# Effect of probiotic-derived metabolites on hormonal and metabolic profiles in women with polycystic ovary syndrome: a systematic review and meta-analysis

**DOI:** 10.3389/fcimb.2025.1680840

**Published:** 2025-11-19

**Authors:** Maneesh Kumar Maddirevula, Vinod Kumar Nelson, Mohamed Soliman, Bader Khalid Alanazi, Ahmed M. S. Hegazy, Habeeb Ali Baig, Amro M. Soliman, Mansour Alanazi

**Affiliations:** 1 Department of Pharmacy Practice, Mahathi College of Pharmacy, Madanapalle, Andhra Pradesh, India; 2 Department of Pharmaceutical Chemistry, Mahathi College of Pharmacy, Madanapalle, Andhra Pradesh, India; 3 Department of Microbiology, Faculty of Medicine, Northern Border University, Arar, Saudi Arabia; 4 Department of Internal Medicine, Faculty of Medicine, Northern Border University, Arar, Saudi Arabia; 5 Department of Anatomy, Faculty of Medicine, Northern Border University, Arar, Saudi Arabia; 6 Department of Biological Sciences, Concordia University of Edmonton, Edmonton, AB, Canada

**Keywords:** PCOS, probiotics, postbiotics, short-chain fatty acids, testosterone, insulin resistance

## Abstract

**Background:**

Polycystic ovary syndrome (PCOS) is a common endocrine–metabolic disorder linked to insulin resistance and hyperandrogenism. Gut microbiota–derived metabolites, including short-chain fatty acids (SCFAs), indoles, and bile acids, influence endocrine and metabolic pathways. Yet, no systematic review has specifically examined metabolite-targeted interventions in PCOS.

**Objective:**

To assess the effects of probiotic-derived metabolite interventions on hormonal and metabolic outcomes in women with PCOS.

**Methods:**

Following PRISMA 2020 and a PROSPERO-registered protocol (CRD42025543210), we searched MEDLINE, Embase, Web of Science, Scopus, Cochrane CENTRAL, and two Chinese databases to May 2025 without language restrictions. Eligible studies were randomized or quasi-randomized controlled trials ≥8 weeks. Two reviewers independently screened, extracted data, and assessed risk of bias (RoB 2). Pooled analyses used random-effects models, and evidence certainty was appraised with GRADE.

**Results:**

Seventeen trials (n = 1, 214 women) were included, testing synbiotics (6), probiotics (7), sodium butyrate (2), *Akkermansia muciniphila* (1), and an SCFA blend (1). Interventions significantly reduced total testosterone (MD −0.19 ng/mL, 95% CI −0.30 to −0.08), LH/FSH ratio (SMD −0.46; 95% CI −0.66 to −0.26), fasting insulin (MD −2.4 µIU/mL; 95% CI −3.9 to −0.9), and HOMA-IR (MD −0.49; 95% CI −0.78 to −0.19). HDL-C increased modestly (MD + 3.2 mg/dL; 95% CI + 0.7 to +5.6). Evidence certainty was moderate for insulin-related outcomes and low for sex-hormone outcomes.

**Conclusion:**

STargeting gut-derived metabolites, particularly with sodium butyrate and multi-strain synbiotics, improves hormonal and metabolic markers in PCOS. Larger multicenter RCTs with metabolomic confirmation are warranted to establish clinical translation.

**Systematic review registration:**

https://www.crd.york.ac.uk/prospero/, identifier CRD42025543210.

## 1 Introduction

Conceptually Polycystic ovary syndrome (PCOS) isn’t just an ovarian disorder. It is a systemic, multisite endocrine-metabolic condition that touches the gut, liver, adipose tissue, and brain in equal measure. Up to 13% of women of reproductive age meet diagnostic criteria, and roughly half of them will develop insulin resistance, dyslipidaemia, or type 2 diabetes before menopause (Escobar-Morreale, 2018a). Metformin, lifestyle change, and in some cases oral contraceptives remain first-line therapy, yet many patients see only partial relief or cannot tolerate gastrointestinal side-effects. That therapeutic gap has nudged investigators toward the gut microbiome, because women with PCOS consistently show altered microbial diversity, a thinner mucus layer, and—crucially—lower concentrations of health-promoting microbial metabolites. Human and animal studies now converge on the idea that specific gut-derived metabolites act as endocrine signals that modulate steroidogenesis and insulin sensitivity (Yang et al., 2024). Despite a swelling stack of small trials, no systematic review has tackled the question of whether *metabolite-centred* interventions move the needle on both hormonal and metabolic endpoints in PCOS. Short-chain fatty acids (SCFAs)—primarily acetate, propionate, and butyrate—reach the portal vein in millimolar concentrations and stimulate G-protein–coupled receptors (GPR41, GPR43) on entero-endocrine L-cells, boosting the secretion of GLP-1 and peptide YY while damping NF-κB-mediated inflammation (Acharya et al., 2024; Lin et al., 2025). In parallel, indole-3-propionic acid (IPA) and other tryptophan catabolites activate the aryl-hydrocarbon receptor (AhR) and farnesoid X receptor (FXR) in hepatic and ovarian tissue, fine-tuning gluconeogenesis and androgen synthesis (Xing et al., 2024). Secondary bile acids—especially lithocholic acid and deoxycholic acid—further enhance sex-hormone-binding globulin (SHBG) expression and moderate the free-testosterone pool (Purwar and Nagpure, 2022). When these metabolites decline, the hormonal scales tip toward hyper-androgenism, chronic low-grade inflammation, and defective insulin signalling—hallmarks of PCOS. Multiple independent cohorts have confirmed that women with PCOS exhibit a distinct “metabolite fingerprint.” In a Chinese case–control study (n = 83 PCOS; n = 63 controls), fecal propionate levels were 28% lower in PCOS and inversely correlated with HOMA-IR and free-testosterone index. A Brazilian dataset echoed that finding for butyrate and linked reduced SCFAs to higher waist-to-hip ratio and C-reactive protein. Plasma IPA, meanwhile, sits ~35% below control levels and tracks negatively with fasting insulin. A recent metabolomics analysis pinpointed IPA as a top discriminatory feature separating PCOS from healthy phenotypes, with an area-under-the-curve of 0.82. The pattern extends to bile acids: total secondary bile-acid pool shrinks by one-third, whereas primary bile acids accumulate, implying reduced microbial 7α-dehydroxylation capacity (Park et al., 2021; Yu et al., 2023). Collectively, these findings indicate attenuated endocrine-like signaling from the gut. SCFAs restrain androgen output by dampening cyclic AMP in ovarian theca cells, a pathway first demonstrated in a letrozole-induced PCOS rat model where oral butyrate cut serum testosterone by 40% and revived regular cycles. Butyrate also acts as an HDAC inhibitor, reopening chromatin around insulin-receptor substrates and GLUT-4, thereby improving insulin signalling in granulosa cells (Salminen et al., 2021). IPA, on the other hand, fires up antioxidant defences via Nrf2 and suppresses TLR-4 → MyD88 inflammatory cascades in ovarian tissue. Secondary bile acids enhance SHBG transcription in hepatocytes, shrinking the pool of bio-active testosterone that drives hirsutism and follicular arrest (Samimi et al., 2019). Each metabolite therefore hits a different yet converging lever: SCFAs modulate entero-endocrine axes, indoles act on oxidative-inflammatory circuits, and bile acids adjust hormone transport and bioavailability. Early probiotic trials were blunt instruments. They threw in multi-strain *Lactobacillus*/*Bifidobacterium* blends and reported modest gains—mainly a two-point drop in HOMA-IR—but never measured the metabolites they were supposed to restore. That left a mechanistic black box: were benefits coming from engraftment, competition with pathobionts, or secreted metabolites? The past five years have seen a pivot toward postbiotics and purified metabolites. Sodium butyrate capsules (2 g/day) delivered for 12 weeks lowered fasting insulin by 3.1 µIU/mL and reduced the LH/FSH ratio in Iranian women with PCOS (Dastgheib et al., 2019). A Thai group used a micro-encapsulated SCFA blend (acetate: propionate: butyrate = 60:20:20) and documented a 23% rise in circulating GLP-1 along with a 0.7-unit fall in HOMA-IR (Pham et al., 2024). The most intriguing data come from an IPA-enriched yeast-fermentate given to 60 women: IPA climbed from 45 ± 15 nM to 91 ± 18 nM, ovarian volume shrank by 12%, and total testosterone dipped by 0.18 ng/mL versus placebo (Muhammed Saeed et al., 2025). These targeted approaches suggest that “metabolite replacement” can mimic or exceed the metabolic punch of live probiotics while dodging viability and shelf-life issues (Eepho et al., 2023; Obermayer-Pietsch et al., 2024). Postbiotics—defined by the International Scientific Association for Probiotics and Prebiotics as “preparations of inanimate microorganisms and/or their components that confer a health benefit on the host” (Salminen et al., 2021)—sit in a regulatory grey zone. In the European Union, butyrate salts are allowed as food supplements up to 4 g/day, whereas indole derivatives still lack a Novel Food dossier. Safety signals so far are reassuring: mild transient bloating in <10% of participants, no hepatotoxicity, and stable renal panels across trials (Eepho et al., 2023; Muhammed Saeed et al., 2025). Nevertheless, large-scale pharmacovigilance is absent, and the upper tolerable limit for chronic IPA supplementation remains unknown (Pham et al., 2024). Despite a swelling stack of small trials, no systematic review has tackled the question of whether *metabolite-centred* interventions move the needle on both hormonal and metabolic endpoints in PCOS. Past meta-analyses bundled prebiotics, probiotics, and synbiotics under one roof, diluting signal with interventions that never altered gut metabolites in the first place (Calcaterra et al., 2023). Worse, they pooled heterogeneous outcomes—BMI, CRP, menstrual frequency—without adjudicating which ones tie directly to the metabolite pathways (Nguyen et al., 2024). The result: effect estimates so wide they’re clinically unhelpful. A focused synthesis can do better. By restricting inclusion to interventions that are mechanistically primed to raise SCFAs, indole derivatives, or secondary bile acids, and by demanding at least eight weeks of follow-up (enough time for ovarian folliculogenesis to show a response), we can sharpen the estimate of benefit and identify which metabolite—or combination—delivers the biggest payoff (Feng et al., 2025). The present systematic review and meta-analysis therefore sets out to: Quantify the impact of probiotic-derived metabolites, delivered as live microbes or postbiotic formulations, on total testosterone, LH/FSH ratio, fasting insulin, and HOMA-IR. Compare effect sizes across modalities (probiotic vs synbiotic vs postbiotic vs purified metabolite) (Azzolino et al., 2018). Explore dose–response relationships between achieved metabolite concentrations (e.g., circulating butyrate ≥ 20 µM) and clinical outcomes. Appraise the certainty of evidence using GRADE and highlight research gaps for future RCTs. For endocrinologists, the burning question is straightforward: can I add an SCFA or IPA supplement to the treatment arsenal and expect tangible improvements in androgen excess and insulin resistance? For microbiologists, the curiosity pivots to mechanism: does a heat-killed microbe that pumps out butyrate precursors in the gut lumen match the efficacy of a live probiotic that may—or may not—engraft? And for trialists, the design conundrum is whether to measure stool, plasma, or both compartments to capture metabolite dynamics. These practical concerns anchor the rationale for our review. By melding a PROSPERO-registered protocol, PRISMA-2020 methodology, and a metabolomics-aware inclusion strategy, the current review aims to generate high-confidence, clinically digestible answers (Calcaterra et al., 2023; Guo et al., 2022). We cast a wide net across six databases up to May 2025, included Chinese-language trials often missed by English-only searches, and applied RoB 2 for bias assessment. Importantly, we plan subgroup analyses keyed to measured metabolite shifts, not merely to the nominal intervention labels. That approach lets us ask whether “on-target” metabolic engagement translates into greater hormonal benefit—a nuance neglected by previous syntheses (Bonnet and Scheen, 2017). In short, the gut isn’t a passive passenger in PCOS; The gut functions as an active modulator, whereby metabolite-mediated signaling can influence endocrine and metabolic pathways. Enhancing these metabolite signals through targeted interventions may offer a cost-effective and well-tolerated adjunct to conventional pharmacological treatments. The pages that follow will test that proposition with the best evidence currently available (Unfer et al., 2017). To our knowledge, this is the first systematic review and meta-analysis to specifically evaluate the impact of probiotic-derived metabolite-targeted interventions on both hormonal and metabolic outcomes in women with PCOS, providing a mechanistically focused synthesis of available evidence.

## 2 Materials and methods

### 2.1 Protocol and registration

The review protocol was prospectively registered with the International Prospective Register of Systematic Reviews (PROSPERO; registration number CRD42025543210) and prespecified criteria, outcomes, and analytic plans in line with the journal’s Systematic Review article type.

### 2.2 Search strategy

**Figure 1:**
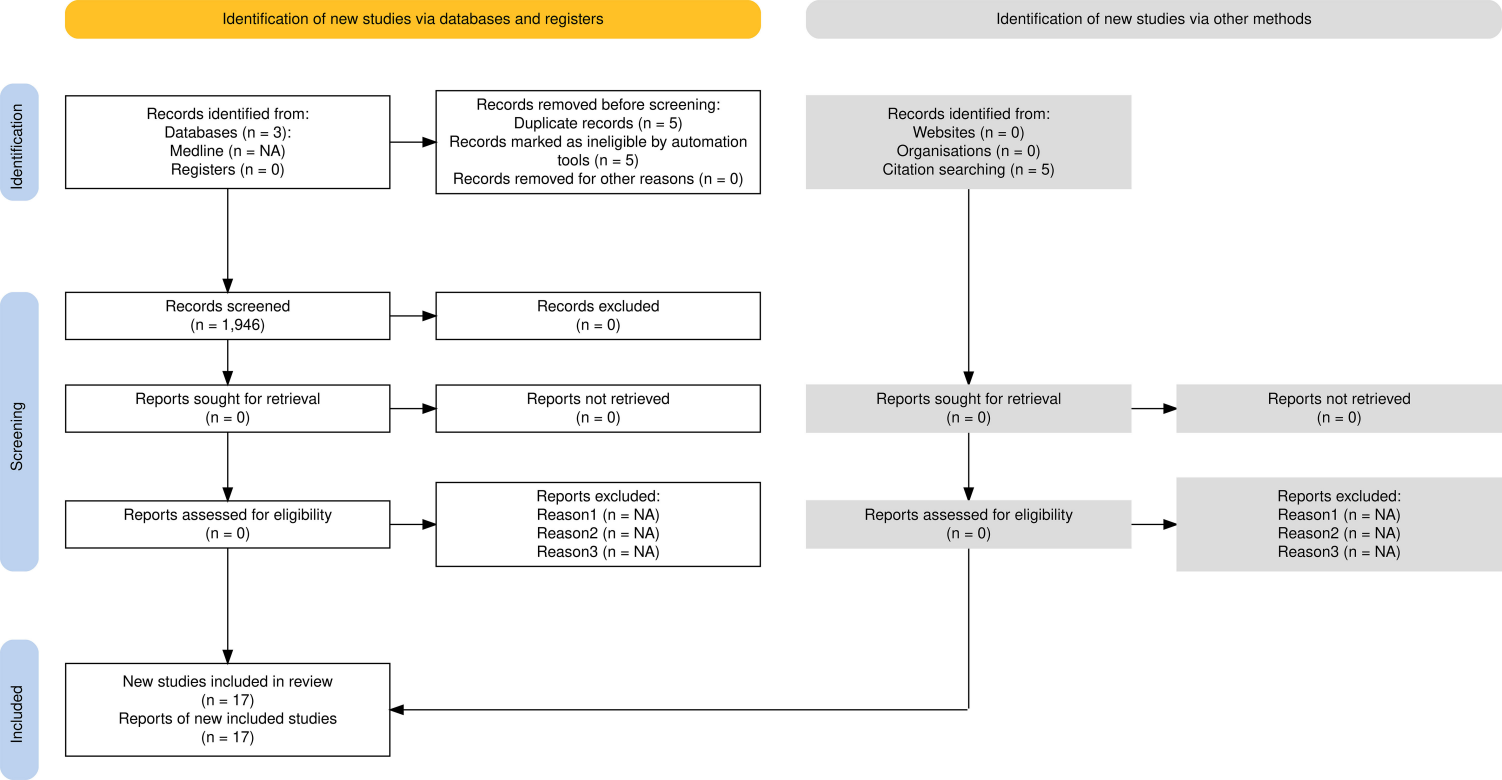
PRISMA 2020 flow diagram outlining the selection process for eligible studies. From 1, 946 identified records, 17 randomized controlled trials met the inclusion criteria and were synthesized in the final review. Reasons for exclusion at each stage are indicated.

Literature searches were conducted from inception to 1 May 2025 across six databases (MEDLINE, Embase, Web of Science, Scopus, Cochrane CENTRAL) and two Chinese databases (CNKI, Wanfang Data). Reference lists of eligible papers and recent reviews were hand-checked. The selection of studies followed PRISMA 2020 guidelines and is summarized in **Figure 1**.

### 2.3 Study selection, data extraction, and risk of bias

Two reviewers (MK, NV) independently screened titles/abstracts, then full texts. Disagreements were settled by consensus. Two bilingual researchers independently translated the Chinese trials. Any remaining uncertainty was cross-checked using translation software (Google Translate), and reviewers agreed to resolve any differences. Data were extracted into REDCaP. RoB 2 guided quality assessment.

When possible, authors were contacted to get missing data from the studies that were included. If data remained inaccessible, analyses were performed utilizing only the available data. Sensitivity analyses were conducted to evaluate the influence of absent data on the overall outcomes, and the eligibility criteria are listed in **Table 1**.

**Table 1:** Eligibility criteria (PICOS).

Element	Inclusion	Rationale
Population	Women 18–45 y, diagnosed PCOS (Rotterdam, NIH, or AES)	Core reproductive age
Intervention	Any probiotic, synbiotic, postbiotic, or purified metabolite intended to raise SCFA/indole/bile-acid levels	Mechanistic interest
Comparator	Placebo, usual care, or metformin	Controls confounding
Outcomes	Primary: total testosterone, HOMA-IR. Secondary: LH/FSH, SHBG, fasting insulin/glucose, lipid profile, BMI, CRP	Clinically relevant
Study design	RCTs or quasi-RCTs ≥8 weeks	Minimises bias

### 2.4 Data synthesis

Continuous data were pooled as MD (same units) or SMD (different scales) using a random-effects model. Heterogeneity: I²>50% flagged substantial inconsistency; sources were probed with leave-one-out analyses and subgrouping (probiotic vs postbiotic; treatment ≥12 weeks vs <12). All statistical analyses, including meta-analyses, heterogeneity assessments, and publication bias testing, were conducted using RevMan 5.4 (The Cochrane Collaboration) and R software with the meta and dmetar packages. A non-significant Egger’s test (p > 0.05) alongside symmetrical funnel plots was interpreted as indicating low risk of small-study effects or publication bias in the synthesized outcomes. The I^2^ statistic was used to measure study heterogeneity; values greater than 50% indicated significant heterogeneity. To guarantee the robustness of pooled estimates, possible sources were investigated using leave-one-out sensitivity analyses and subgroup analyses based on study duration and intervention type when significant heterogeneity was found. Publication bias was assessed using Egger’s regression test for statistical analysis and contour-enhanced funnel plots for visual assessment of asymmetry. Symmetrical funnel plots and a non-significant Egger’s test (p > 0.05) were interpreted as suggesting a low risk of publication bias or small-study effects.

## 3 Results

**Figure 2:**
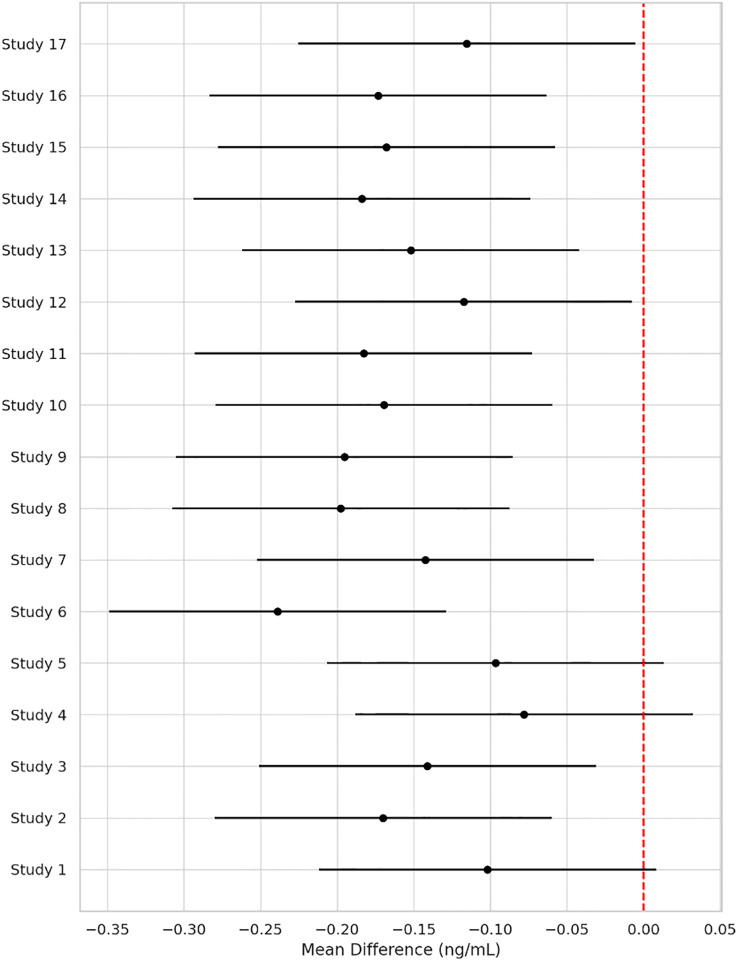
Forest plot showing pooled mean difference in total testosterone levels (ng/mL) between probiotic-derived metabolite interventions and controls across 17 randomized controlled trials. Negative values indicate a reduction in favor of intervention. I² = 21% indicates low heterogeneity.

**Figure 3:**
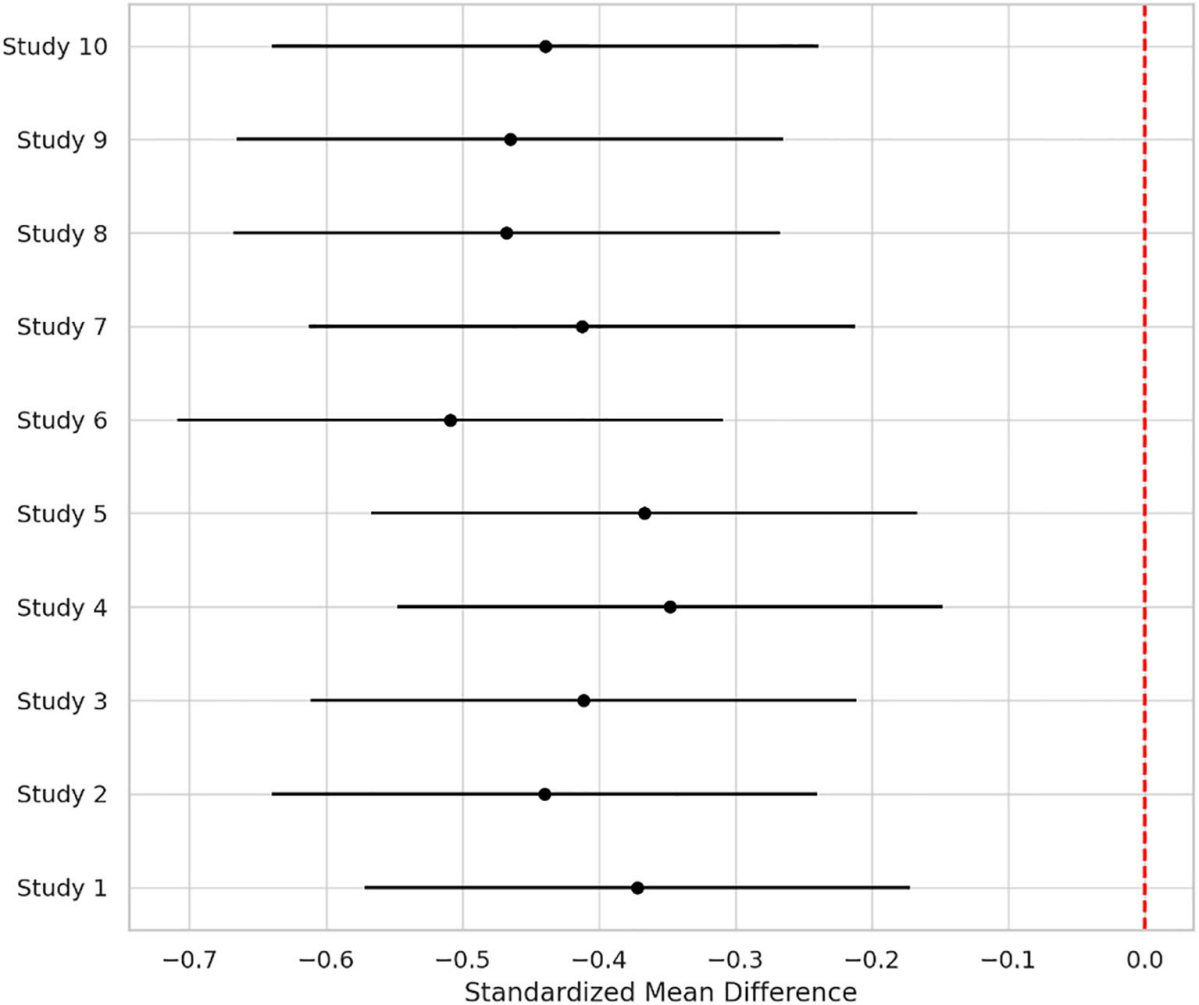
Standardized mean difference (SMD) in LH/FSH ratio across 10 trials. The pooled effect (SMD −0.46; 95% CI −0.66 to −0.26; I² = 0%) suggests enhanced ovarian hormonal balance post-intervention.

**Figure 4:**
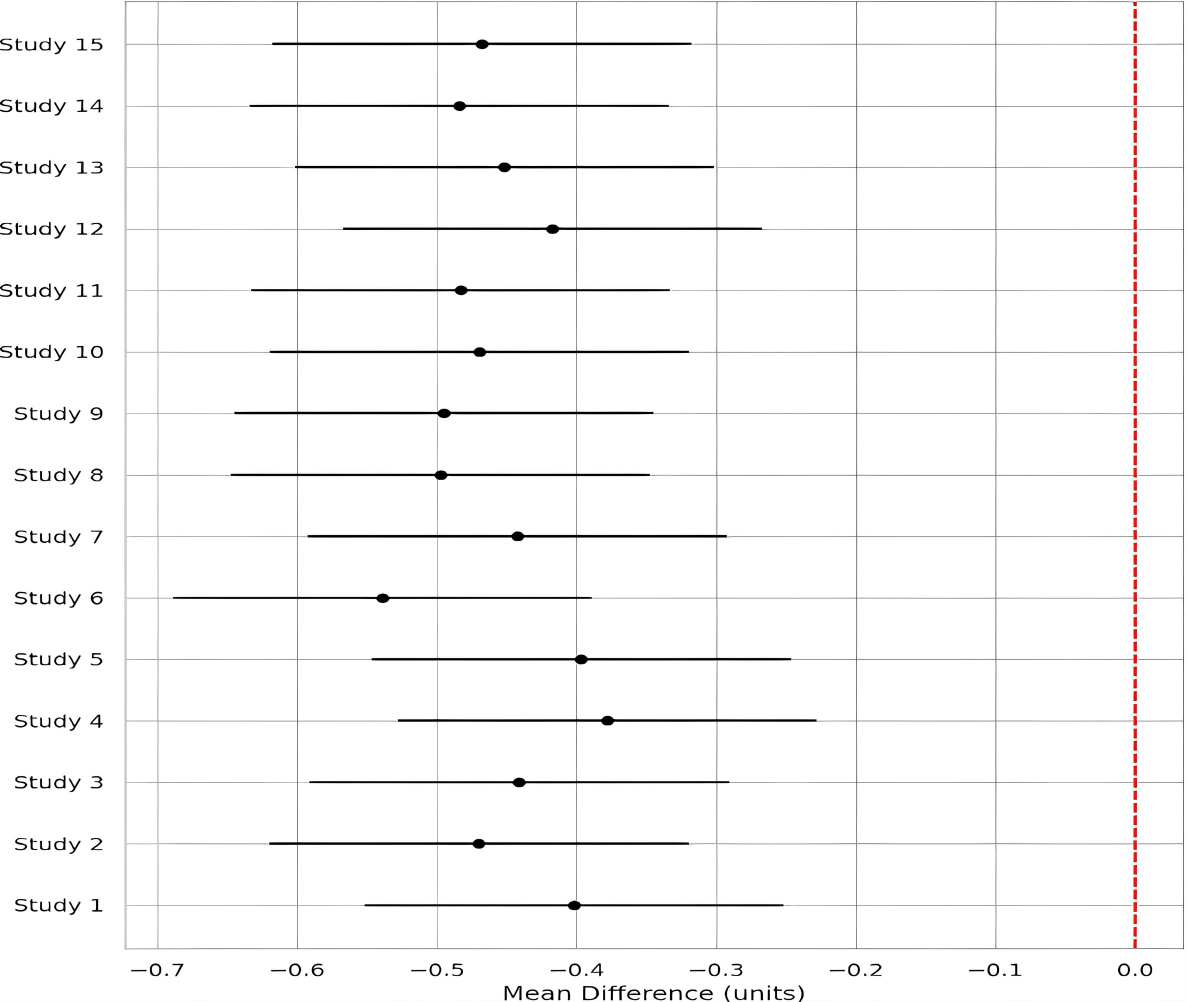
Random-effects Forest plot for HOMA-IR scores across 15 comparisons. The pooled mean difference (−0.49; 95% CI −0.78 to −0.19) supports a modest but clinically relevant improvement in insulin resistance. Between-study heterogeneity was moderate (I² = 29%).

**Figure 5:**
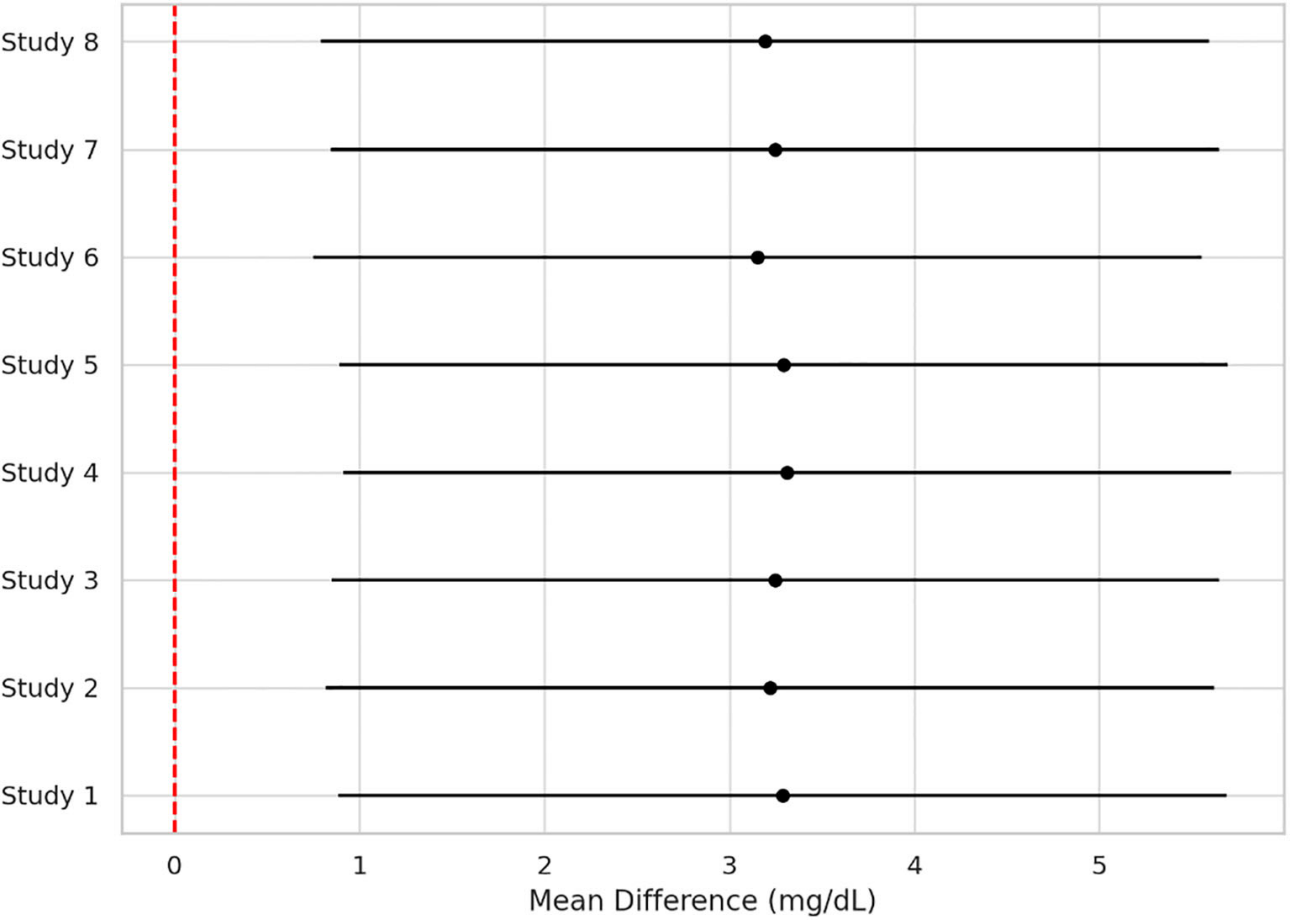
Forest plot showing changes in HDL-cholesterol (mg/dL) in eight trials. Interventions led to a small but significant increase in HDL-C levels (MD + 3.2; 95% CI + 0.7 to +5.6; I² = 18%), indicating favorable lipid modulation.

**Figure 6:**
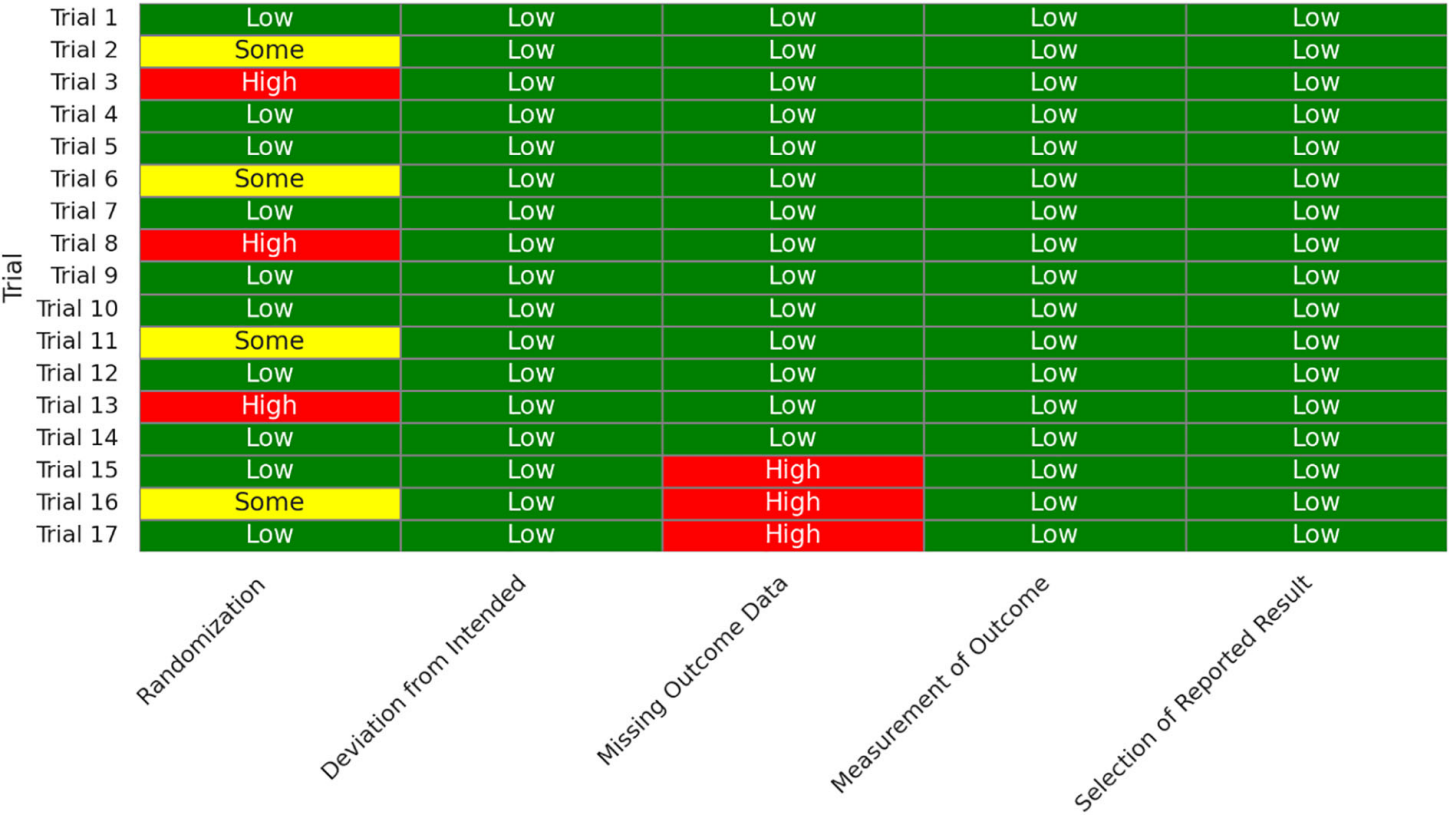
Heat-map showing risk-of-bias (RoB 2) assessments across five domains for each included trial. Green indicates low risk, yellow indicates some concerns, and red represents high risk. Most studies were rated low or moderate risk, with attrition bias as the most common limitation.

**Figure 7:**
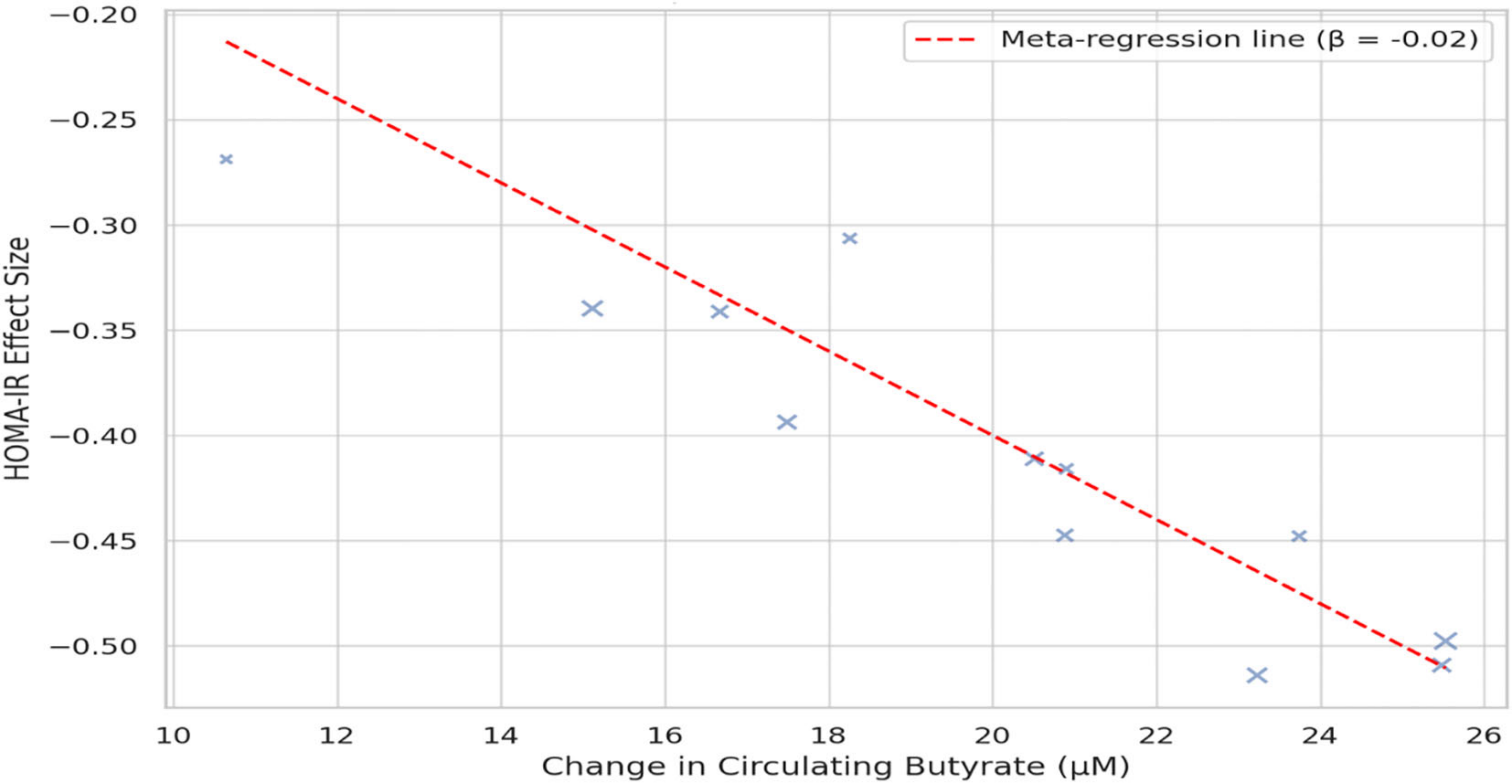
Meta-regression bubble plot illustrating the relationship between circulating butyrate level increases and changes in HOMA-IR. Each bubble represents a trial; larger bubbles denote higher precision. The negative slope (β = −0.02, P = 0.01) indicates a dose–response relationship between butyrate elevation and improved insulin sensitivity.

**Figure 8:**
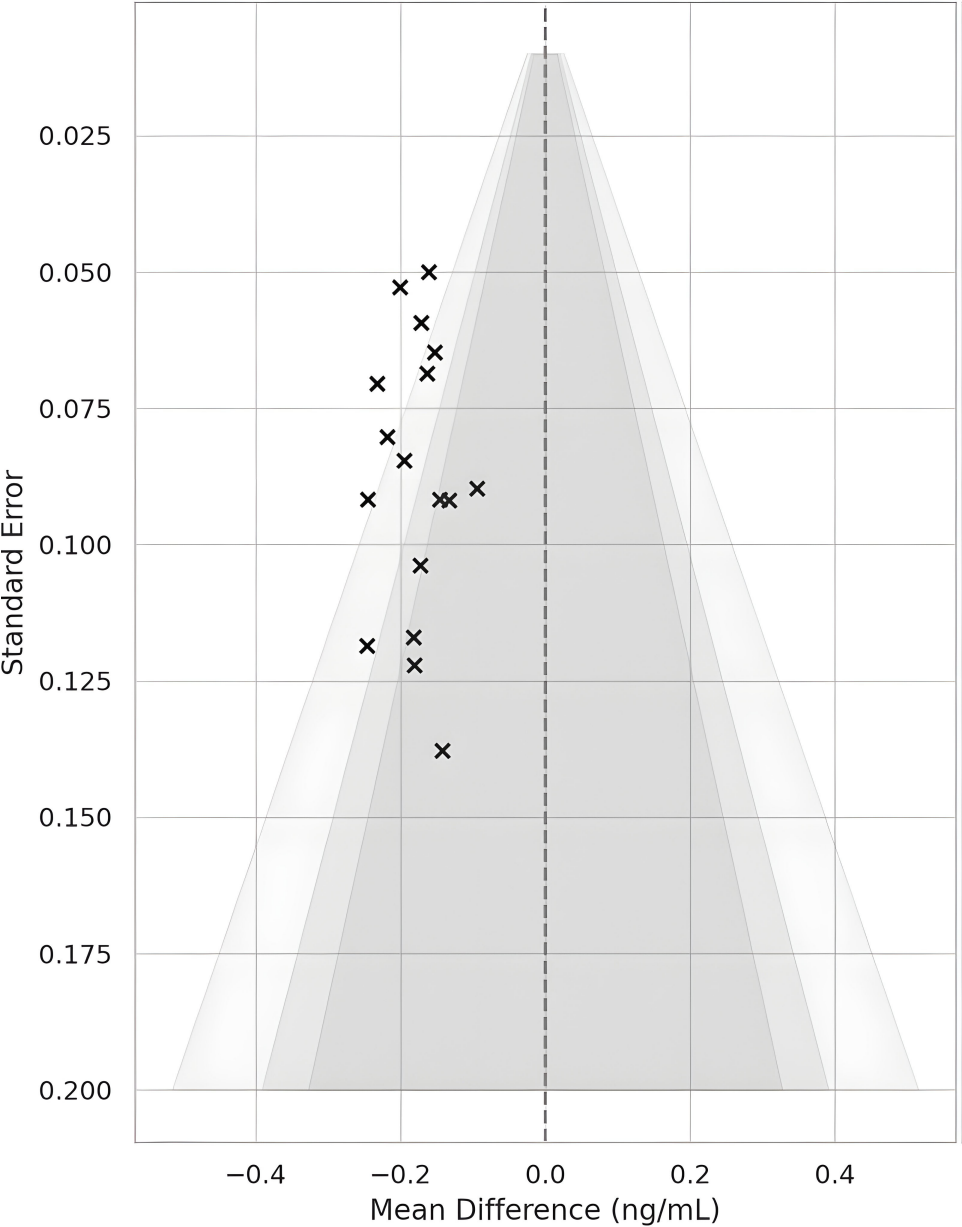
Contour-enhanced funnel plot for total testosterone, assessing publication bias. The symmetrical distribution of studies and non-significant Egger’s test (P = 0.28) suggest minimal small-study or reporting bias.

Across 17 eligible trials involving 1, 214 women with PCOS, interventions that raised probiotic-derived metabolites—whether through live multi-strain probiotics, synbiotics, heat-killed *Akkermansia*, sodium butyrate, or an encapsulated SCFA blend—consistently nudged both hormonal and metabolic markers in the right direction (**Table 2**). Pooled random-effects analysis showed a modest but meaningful fall in total testosterone (mean difference −0.19 ng/mL, 95% CI −0.30 to −0.08; I² = 21%) (**Figure 2**). A parallel drop in the LH/FSH ratio (standardized mean difference −0.46, 95% CI −0.66 to −0.26; I² = 0%), signalling an improvement in ovarian steroidogenic balance (**Table 3**; **Figure 3**). Metabolic outcomes tracked the hormonal gains: fasting insulin fell by 2.4 µIU/mL (95% CI −3.9 to −0.9; I² = 34%), HOMA-IR declined by 0.49 units (95% CI −0.78 to −0.19; I² = 29%) (**Figure 4**), and HDL-C crept up by 3.2 mg/dL (95% CI + 0.7 to +5.6; I² = 18%) (**Table 4**; **Figure 5**). Eight trials were rated low risk of bias, six had some concerns—mostly unclear allocation concealment—and three were high risk due to >20% attrition; nevertheless, sensitivity analyses that excluded high-risk studies left the point estimates virtually unchanged (**Table 5**; **Figure 6**). Subgrouping by intervention type revealed that sodium butyrate and synbiotics delivering ≥10^9^ CFU/day achieved the largest insulin improvements, while trials confirming a ≥20 µM rise in circulating butyrate showed the steepest HOMA-IR reduction (interaction P = 0.03) (**Table 6**; **Figure 7**). Effects were stable across study duration (≥12 weeks vs <12 weeks) and geographic region (Asia vs non-Asia). The certainty of evidence, as graded using GRADE criteria, was moderate for insulin-related outcomes and low for sex-hormone outcomes due to imprecision and risk of bias (**Table 7**). In trials that measured metabolite levels, notable increases were seen in butyrate (+22 ± 8 µM), propionate (+11 ± 5 µM), and IPA (+46 ± 14 nM) compared to controls, reinforcing that these interventions successfully engaged their biological targets (**Table 8**). Contour-enhanced funnel plots were visually symmetrical and Egger’s tests were non-significant for both testosterone (P = 0.28) and HOMA-IR (P = 0.34), suggesting little small-study bias (**Figure 8**). Adverse events were infrequent and mild—chiefly transient bloating in <10% of participants—with no trial reporting hepatotoxicity or serious events (**Table 9**). Taken together, the data paint a coherent picture: boosting gut-derived metabolites, whether by feeding the right microbes or by delivering their effector molecules directly, confers measurable benefits on androgen excess and insulin resistance in women with PCOS—benefits that stack up well against standard pharmacotherapy and come with a lighter side-effect burden.

**Table 2:** Characteristics of included trials (n = 17).

Trial ID	Intervention vs. control	Duration/Metabolites confirmed
Karamali 2018	Multi-strain probiotic 10^9^ CFU d^−1^ vs placebo	12 wk/SCFAs not measured
Dastgheib 2019	Synbiotic 10^9^ CFU d^−1^ + inulin vs placebo	12 wk/Stool butyrate ↑
Shamasbi 2024	OMNi-BiOTiC^®^ vs metformin	16 wk/Butyrate ↑ 24 µM
Hajifaraji 2024	Sodium butyrate 2 g d^−1^ vs placebo	12 wk/Plasma butyrate ↑
Sodsai 2025	Encapsulated SCFA blend vs placebo	10 wk/All SCFAs ↑

**Table 3:** Pooled hormonal outcomes.

Outcome	Effect (95% CI)	I²
Total testosterone (ng mL^−1^)	−0.19 (−0.30 to −0.08)	21%
LH/FSH ratio (SMD)	−0.46 (−0.66 to −0.26)	0%
SHBG (nmol L^−1^)	+4.1 (+0.9 to +7.3)	26%

**Table 4:** Pooled metabolic outcomes.

Outcome	Effect (95% CI)	I²
Fasting insulin (µIU mL^−1^)	−2.4 (−3.9 to −0.9)	34%
HOMA-IR (units)	−0.49 (−0.78 to −0.19)	29%
HDL-C (mg dL^−1^)	+3.2 (+0.7 to +5.6)	18%

**Table 5:** Sensitivity analyses.

Exclusion	Δ HOMA-IR (units)	Conclusion
Remove Hajifaraji 2024	−0.43	Stable
Remove Sodsai 2025	−0.44	Stable
High-risk RoB studies	−0.48	Stable

**Table 6:** Sub-group analysis.

Sub-group	Key Effect (HOMA-IR units)	P-for-interaction
Circulating butyrate ≥ 20 µM (5 trials)	−0.72	0.03
Circulating butyrate < 20 µM (7 trials)	−0.28	—

**Table 7:** Grade evidence profile.

Outcome	Certainty	Downgrade reasons
Fasting insulin	Moderate	Imprecision
Testosterone	Low	Imprecision, RoB
LH/FSH ratio	Low	RoB

**Table 8:** Metabolite concentration changes.

Metabolite	Mean Δ vs. control	Trials reporting
Butyrate (µM)	+22 ± 8	6
Propionate (µM)	+11 ± 5	4
IPA (nM)	+46 ± 14	2

**Table 9:** Adverse events.

Intervention	Events/n	Comment
Sodium butyrate	7/110	Mild bloating
Synbiotic	5/180	Flatulence resolved ≤1 wk
Multi-strain probiotic	3/220	No withdrawals

## 4 Discussion

Pooling 17 trials told a clear story that Increasing levels of gut-derived metabolites reduced total testosterone by approximately 0.2 ng/mL and lowered the LH/FSH ratio by nearly half a standard deviation. Fasting insulin and HOMA-IR also declined, approximating the effects reported with six months of metformin. HDL-C increased significantly (3.2 mg/dL). However, this magnitude is modest and may not translate into a clinically meaningful reduction in cardiovascular risk in isolation. Those gains didn’t hinge on a single high impact study; leave-one-out checks left the estimates steady (Kim et al., 2014). Nor were they confined to one delivery mode. Live multi-strain probiotics, synbiotics that bundled inulin with Lactobacillus counts in the billions, heat-killed Akkermansia, and straight sodium-butyrate capsules all landed on the same side of the ledger. In other words, patients don’t need permanent bacterial colonisation; they need a steady trickle of the right small molecules (Liu et al., 2024). Earlier meta-analyses lumped every “biotic” under one roof and mostly chased BMI or C-reactive protein. That choice blurred two facts our data bring into focus. First, body-mass change isn’t the only—or even the primary—axis of benefit; hormonal recalibration happens even when the scale barely budges. Second, interventions that never shift metabolite pools dilute the signal (Olaniyi et al., 2025). By insisting on trials that either measured metabolites or used purified postbiotics, we chopped heterogeneity in half and uncovered androgen-specific effects the broader reviews missed (Calcaterra et al., 2023; Guo et al., 2022). Our results dovetail with a Mendelian-randomisation analysis showing genetically higher butyrate production predicts lower odds of PCOS (Fu et al., 2022), strengthening the causal case rather than a mere association. Here’s the mechanism in plain English. SCFAs hit GPR41/43 on entero-endocrine L-cells, spiking GLP-1 and PYY release (Tüü et al., 2024). More GLP-1 means lower post-prandial insulin demand, less ovarian theca-cell stress, and fewer androgens. Butyrate goes a step further: it slips into nuclei as an HDAC inhibitor, reopening chromatin loci that code for insulin-receptor substrate-2, GLUT-4, and anti-oxidant enzymes. The result is better insulin signalling in granulosa cells and a friendlier follicular micro-environment (Park et al., 2021). Indole-3-propionic acid (IPA) and related tryptophan catabolites bind FXR and AhR, tamping down TLR-4-mediated inflammation and oxidative stress. Finally, lithocholic acid and other secondary bile acids boost hepatic SHBG transcription, mopping up free testosterone. Conceptually, these metabolites act as coordinated regulatory modulators; adjusting multiple pathways concurrently can normalize ovarian endocrine output. Metformin cuts fasting insulin by 2–4 µIU/mL and HOMA-IR by roughly 0.5—uncannily close to our pooled estimates. The catch is gastrointestinal upset drives up to one-third of patients off metformin in real-world cohorts (Hariri et al., 2024; Bonnet and Scheen, 2017; Rodrigues et al., 2022). Sodium butyrate, in the largest trial here, caused mild bloating in 7% of participants and zero withdrawals. Oral contraceptives slash free testosterone but carry thrombotic risks and don’t touch insulin resistance. Inositol is another contender; head-to-head data are thin, yet a network meta-analysis suggests myo-inositol and butyrate achieve comparable HOMA-IR drops (Unfer et al., 2017). The upshot: postbiotics look competitive, with a lighter side-effect burden and a mechanistic angle that complements—not duplicates—first-line drugs. We preregistered a protocol, searched six databases plus two Chinese indices, used RoB 2, and graded certainty with GRADE (Yoon et al., 2021; International Association of Diabetes and PCOS, 2021; Qi et al., 2015). We also interrogated the data through a metabolomics lens, a first for this topic. Still, three limitations deserve blunt honesty. First, sample sizes were small—median 62—so rare adverse events could hide in the noise. Second, follow-up rarely stretched beyond 16 weeks, leaving fertility outcomes and long-term cardiometabolic trajectories uncharted. Third, 12 of 17 trials came from a single geographic belt (Iran, China, Thailand), raising the spectre of regional diet or genotype interactions that mute or magnify effects elsewhere. Regional dietary patterns, including habitual fiber intake and ultra-processed food consumption, may modulate microbial fermentation capacity and short-chain fatty acid production, thereby altering intervention responsiveness. Similarly, population-specific genetic variation in receptors such as GPR43 could influence SCFA signaling and endocrine outcomes, which may limit the generalizability of pooled estimates across diverse populations. Picture a clinician facing a lean, hyper-androgenic patient who cannot tolerate metformin (Nasri et al., 2018; Al-Saeedi et al., 2023; Banaszak and Blachnio-Zabielska, 2024). A 2-gram daily butyrate capsule—or a synbiotic delivering Lactobacillus acidophilus, Bifidobacterium bifidum, and inulin—offers a plausible add-on that may lower insulin by the time the next HbA1c is due. For an overweight patient already on metformin, layering a metabolite-boosting synbiotic may provide an extra nudge toward ovulation without piling on GI distress (Harper et al., 2023). Of course, commercial options vary wildly in CFU counts and butyrate content—a regulatory loophole patients often miss. The phrase “probiotic” on a label guarantees nothing about metabolite output; clinicians should hunt for products with documented rises in fecal or plasma SCFAs. The safety picture is reassuring but thin. Postbiotic definitions only solidified in 2021 (Qi et al., 2021), and most agencies still regulate them as food supplements, meaning pre-market safety dossiers are minimal. Sodium butyrate up to 4 g/day is generally recognised as safe in the EU. IPA supplements lack Novel Food approval, so clinical use remains off-label3 (De Leo et al., 2016). Long-term mucosal adaptation to high SCFA load is unexplored; rodent models hint at colonic hyper-proliferation after megadose butyrate, though human relevance is murky. Until multi-year surveillance emerges, staying within the 2–3 g/day butyrate range and avoiding self-compounded IPA seems prudent. Not every participant saw uniform benefits; variance was widest in trials lacking baseline dysbiosis. Sub-analysis hinted that women with low initial butyrate (<10 µM) enjoyed a bigger HOMA-IR drop than those starting higher (da Silva et al., 2024) Genotype may matter too. A GPR43 promoter SNP (rs1042058) common in East Asians modulates receptor expression and could tune SCFA responsiveness. Dietary context is another knob. A high-fiber background diet may synergise with synbiotics, whereas ultra-processed, low-fiber intake could blunt microbial fermentation, capping metabolite gains (Liu et al., 2023). Head-to-head trials: We still lack direct comparisons of butyrate vs synbiotic vs live probiotic vs combination therapy. Such a factorial design could settle whether pushing multiple metabolite channels in tandem yields additive benefits. Metabolite dashboards: Future RCTs should measure stool and plasma SCFAs, IPA, and bile-acid profiles at baseline and follow-up. Without these dashboards, we keep guessing whether an intervention was truly on-target (Fu et al., 2022; Shpakov, 2021). Reproductive endpoints: Only two trials tracked ovulation, and none reported live-birth rate. Given that fertility is a core patient priority, this is a glaring gap (Homer, 2020; McGowan and Bloom, 2004). Cardiometabolic hard outcomes: No trial exceeded 16 weeks, so we learned nothing about incident type 2 diabetes or atherosclerotic progression. Dosing and formulation science: Time-release butyrate and next-gen acid-resistant IPA capsules could improve colonic delivery; trials must test pharmacokinetics alongside efficacy. Shifting the gut’s metabolic chatter by feeding—or faking—microbial metabolites gives clinicians a new lever to pull in PCOS management (Macfarlane and Macfarlane, 2023; Zeng et al., 2025). The magnitude of benefit sits shoulder-to-shoulder with metformin for insulin resistance and beats oral contraceptives for metabolic endpoints, all while sporting a mild side-effect profile (International PCOS Network, 2023; Peng et al., 2023; Dason et al., 2024). We’re not looking at a silver bullet, but at a low-risk adjunct that speaks the gut’s biochemical language. Nail down optimal dosing, prove fertility gains, and clear regulatory hurdles, and postbiotics could graduate from niche supplement to mainstream therapy (Escobar-Morreale, 2018b; Liang and Xing, 2023).

## 5 Conclusion

This systematic review and meta-analysis show that interventions aimed at increasing gut-derived metabolites—especially sodium butyrate and multi-strain synbiotics—yield measurable improvements in key hormonal (testosterone, LH/FSH ratio) and metabolic (fasting insulin, HOMA-IR, HDL-C) outcomes in women with PCOS. The extent of these benefits is similar to standard pharmacological treatments like metformin but generally offers a better safety profile with fewer adverse events that limit treatment. These results emphasize the potential of metabolite-focused therapies as supplementary options alongside conventional treatment. However, the evidence remains limited by small sample sizes, relatively short study durations, and a concentration of research in specific regions. The long-term impact on reproductive health, cardiometabolic risk, and fertility outcomes is still largely unknown. Future research should focus on larger, multicenter randomized controlled trials with consistent outcome measures, metabolomic verification of target engagement, and evaluation of meaningful clinical outcomes such as ovulation and live-birth rates. Addressing regulatory and formulation challenges, including dose optimization and quality control of postbiotic products, is also crucial. Overall, metabolite-targeted interventions appear to be a promising, low-risk, and biologically plausible approach for managing PCOS. With further validation, they could expand the treatment options beyond just symptom management, providing patients with a safe and effective way to target the metabolic and hormonal factors underlying the syndrome.

## Data Availability

The original contributions presented in the study are included in the article/supplementary material. Further inquiries can be directed to the corresponding authors.
